# Microalgal photoautotrophic growth induces pH decrease in the aquatic environment by acidic metabolites secretion

**DOI:** 10.1186/s13068-022-02212-z

**Published:** 2022-10-26

**Authors:** Mingcan Wu, Guimei Wu, Feimiao Lu, Hongxia Wang, Anping Lei, Jiangxin Wang

**Affiliations:** 1 Shenzhen Key Laboratory of Marine Bioresource and Eco-Environmental Science, Shenzhen Engineering Laboratory for Marine Algal Biotechnology, Guangdong Provincial Key Laboratory for Plant Epigenetics, College of Life Sciences and Oceanography, Shenzhen University, Shenzhen, 518060 China; 2 State Key Laboratory of Marine Resource Utilization in South China Sea, College of Oceanology, Hainan University, Haikou, 570228 China; 3 Center for Microalgal Biotechnology and Biofuels, Institute of Hydrobiology, Chinese Academy of Sciences, Wuhan, 430072 China

**Keywords:** Microalgae, *Euglena gracilis*, Aquatic environmental pH, Humic acids, Acidic metabolites

## Abstract

**Background:**

Microalgae can absorb CO_2_ during photosynthesis, which causes the aquatic environmental pH to rise. However, the pH is reduced when microalga *Euglena gracilis* (EG) is cultivated under photoautotrophic conditions. The mechanism behind this unique phenomenon is not yet elucidated.

**Results:**

The present study evaluated the growth of EG, compared to *Chlorella vulgaris* (CV), as the control group; analyzed the dissolved organic matter (DOM) in the aquatic environment; finally revealed the mechanism of the decrease in the aquatic environmental pH via comparative metabolomics analysis. Although the CV cell density was 28.3-fold that of EG, the secreted-DOM content from EG cell was 49.8-fold that of CV (*p*-value < 0.001). The main component of EG’s DOM was rich in humic acids, which contained more DOM composed of chemical bonds such as N–H, O–H, C–H, C=O, C–O–C, and C–OH than that of CV. Essentially, the 24 candidate biomarkers metabolites secreted by EG into the aquatic environment were acidic substances, mainly lipids and lipid-like molecules, organoheterocyclic compounds, organic acids, and derivatives. Moreover, six potential critical secreted-metabolic pathways were identified.

**Conclusions:**

This study demonstrated that EG secreted acidic metabolites, resulting in decreased aquatic environmental pH. This study provides novel insights into a new understanding of the ecological niche of EG and the rule of pH change in the microalgae aquatic environment.

**Supplementary Information:**

The online version contains supplementary material available at 10.1186/s13068-022-02212-z.

## Background

Unicellular microalgae are photosynthetic organisms with fast growth and strong stress resistance [1]. Microalgae contribute to more than 40% of global primary biomass production. They are suitable candidates for various biotechnology applications such as food, feed products, drugs, fuels, wastewater treatment, and atmospheric carbon mitigation [1, 2]. At present, the most economic benefit is the outdoors large-scale cultivation of microalgae, as microalgae can accumulate a large amount of biomass by absorbing CO_2_, requiring a small number of nutrients under sunlight conditions [3, 4]. However, the aquatic environmental pH can gradually increase during most microalgae culture [5, 6]. For example, the pH of *Chlorella vulgaris* aquatic environment was enhanced from 6 to above 10 in the later cultured stage. Moreover, the microalgae photosynthetic efficiency could be inhibited after the pH was > 11, thereby reducing the final biomass [7].

The main reason for the increased aquatic environmental pH is that CO_2_ can be dissolved in the aquatic environment to form HCO_3_^−^. HCO_3_^−^ can be quickly passed through the microalgae's carbon concentrating mechanisms (CCMs) with cellular carbonic anhydrases that convert HCO_3_^−^ to CO_2_ and OH^−^ [8]. CO_2_ and H_2_O are fixed into the organic matter and O_2_ by ribulose-1,5-bisphosphate carboxylase/oxygenase (RuBisCO) [8]. To a lesser extent, NO_3_^−^ uptake and reduction (NO_3_^−^ to NH_3_) for amino acid synthesis would also release a part of OH^−^ ions into the aquatic environment. Finally, the excess OH^−^ ions in the cells are released into the aquatic environment, enhancing pH [6]. However, for some economic microalgae such as single-celled *Euglena gracilis* aquatic environmental pH would be reduced when cultured under photoautotrophic conditions. For example, when the initial pH was as low as 3.5 to 2, the biomass of *E. gracilis* gradually increased [9]. This phenomenon is contrary to traditional theory. The mechanism of how these microalgal species decrease the aquatic environmental pH remains unknown.

*Euglena gracilis* is a flagellated, unicellular microalga without a cell wall. It is rich in immunity-improving paramylon, tocopherol with potent antioxidant capacity, and a wide range of amino acids [10, 11]. Moreover, *E. gracilis* has been commercially applied in various fields in Japan [12]. *Euglena* is suitable to survive under acidic water conditions. For example, the photosynthetic microalga *Euglena mutabilis* lives in acid mine drainages and could secrete a large amount of organic matter into the aquatic environment, providing nutrients for different microorganisms and playing an important ecological niche [13]. In addition, *E. gracilis* could secrete succinic acid, lactic acid, and amino acids into the aquatic environment [14, 15]. Moreover, in our previous study, during the pilot-scale fermentation of *E. gracilis*, the rate of NaOH consumption was much higher than that of other microalgae fermentation (e.g., *Chlorella sorokiniana* GT-1, *Scenedesmus acuminatus* GT-2) [16]. These results suggested that *E. gracilis* may be a microalga that easily secretes acidic metabolites into the aquatic environment, causing the pH to be reduced. However, this hypothesis has still not been confirmed.

There are many methods for characterizing dissolved organic matter (DOM) in the aquatic environment, such as three-dimensional fluorescence excitation-emission matrix (3D-EEM) spectra [17] and Fourier transform infrared (FTIR) [18] analysis. These techniques were widely used in *S. acuminatus* [19, 20], *Nannochloropsis oceanica* [21], *E. gracilis* [22]. Their growth inhibitors like humic acid were found in the aquatic environment. In addition, metabolomics refers to studying metabolites (intermediate products) from various metabolic pathways of a specific organism. Qualitative and quantitative metabolomics can reveal changes in metabolic state, detect biomarkers, explicate the study-related metabolic pathways, and determine the mechanisms involved in an organism responding to environmental stimuli [23, 24]. This type of scientific study analyzes multiple metabolic pathways to reveal what best reflects the pathway of a cell. However, most omics are currently only used to analyze the differences in metabolites within cells. The connection between metabolites in the cell and the aquatic environment is rarely reported.

This study took *E. gracilis* as the research object and *C. vulgaris* as the control group. Firstly, evaluate the growth of these two kinds of microalgae; secondly, analyze the DOM in the aquatic environment; finally, reveal the mechanism of the decrease in the aquatic environmental pH by comparative metabolomics analysis. This result affords novel insights into the pH change mechanism and the ecological niche of *E. gracilis* in the aquatic environment.

## Results and discussion

### Photoautotrophic growth and potential maximum photosynthetic capacity of *E. gracilis*

**Fig. 1:**
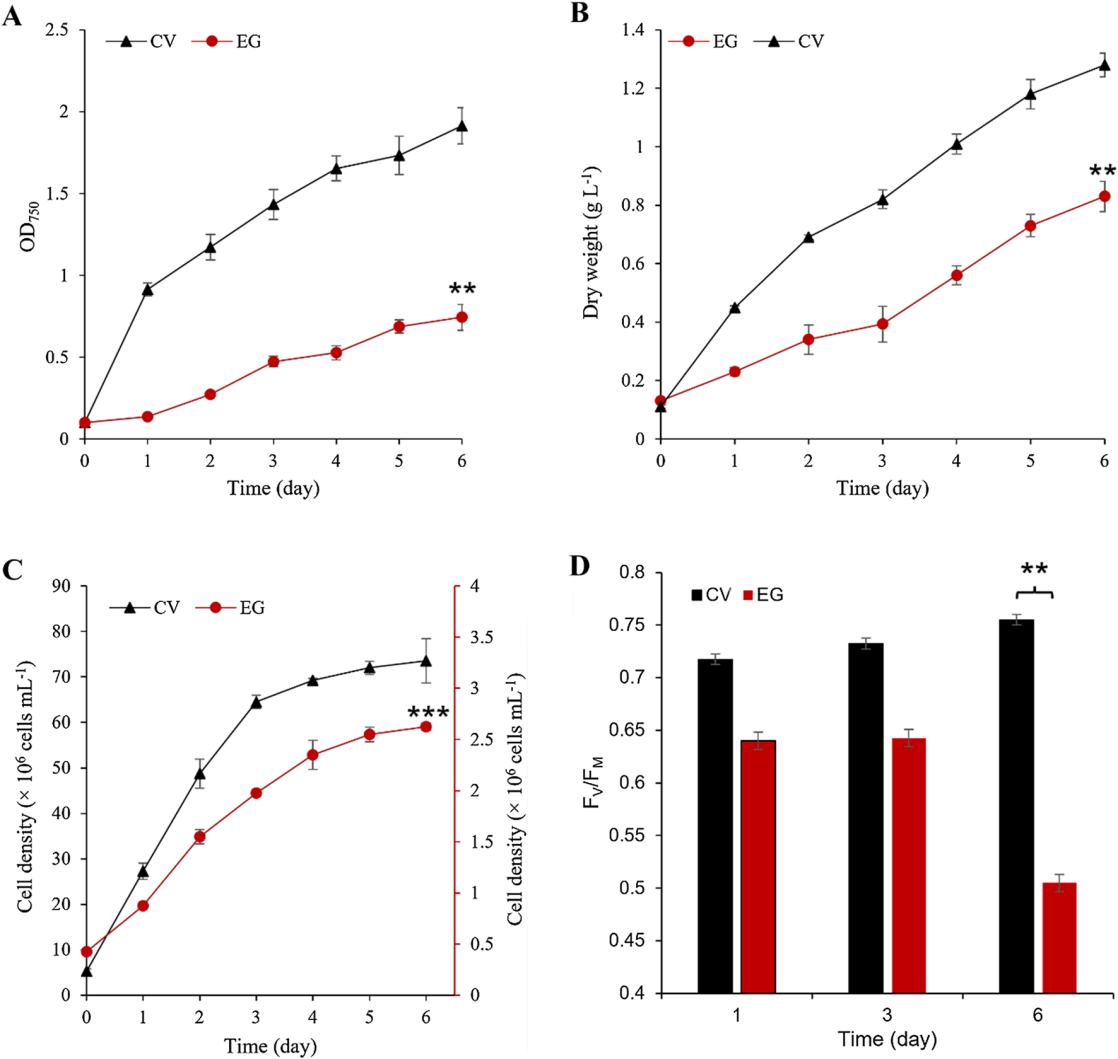
Growth and *F*_v_/*F*_m_ of *E. gracilis* under photoautotrophic conditions compared to *C. vulgaris*. EG represents *E. gracilis*; CV represents *C. vulgaris,* as the control group; **A** OD_750_; **B** dry weight; **C** cell density; **D** *F*_v_/*F*_m_; *F*_v_/*F*_m_ reflects the maximum quantum efficiency of photosystem II (PSII) photochemistry in microalgae. ** represents *p* < 0.01, *** represents *p* < 0.001; the values represent mean ± S.D. *n* = 3

The results showed that the biomass and cell density of *E. gracilis* (EG) and *C. vulgaris* (CV, as a control group) gradually increased under the photoautotrophic conditions (Fig. 1A–C). However, the accumulated biomass of EG was significantly lower than that of CV, especially the CV biomass (OD_750_ = 1.9, 1.3 g L^−1^) was 2.7 (1.5)-fold than that of EG (OD_750_ = 0.7, 0.8 g L^−1^) (Fig. 1A, B, *P* < 0.01) at day 6, respectively. Similarly, the CV cell density (73.6 × 10^6^ cells mL^−1^) was 28.3-fold than that of EG (2.6 × 10^6^ cells mL^−1^) on the 6th day, suggesting that the EG cell density was significantly lower than that of CV (*P* < 0.001) (Fig. 1C). The potential maximum photosynthetic capacity (*F*_v_/*F*_m_) reflects the ability of microalgae to dissipate, absorb, and transmit light energy. It is a valuable parameter that indicates physiological state and growth rate and is also an internal probe of the relationship between microalgae and their environment [20, 25, 26]. The EG *F*_v_/*F*_m_ was lower than CV (*p* < 0.01). For instance, the CV *F*_v_/*F*_m_ (0.8) was 1.6-fold that of EG (0.5) on day 6 (Fig. 1D, *p* < 0.01), suggesting that EG potential carbon fixation capacity was lower than that of CV, which may also explain why the biomass of EG was lower than CV. Usually, the chloroplast is the place where cells carry out photosynthesis. According to literature reports, EG chloroplasts were derived from secondary endosymbiotic green microalgae [25]. Its chloroplasts were easily permanently lost after being treated by the external chemicals, such as erythromycin [27], and ofloxacin [28], thereby reducing the photosynthetic efficiency of EG. Moreover, it can trigger EG trophic type from the original photoautotrophic type (CO_2_ as a carbon source) to heterotrophic (organic matter, e.g., glucose as a carbon source) type. In addition, *E. gracilis* *F*_v_/*F*_m_ can be reduced via secreted humic acid under recycled-cultured conditions [22], suggesting that a part of the biomass accumulated by *E. gracilis* may be converted into secreted metabolites through photosynthesis so that the accumulated biomass becomes lower. Especially when these secreted metabolites were secreted into the aquatic environment and then became humic acid, which led to a decrease in EG *F*_v_/*F*_m_, which may further inhibit EG photosynthetic efficiency. Therefore, we speculated that EG might secrete more growth-inhibiting organics than CV, reducing EG photosynthetic efficiency.

### *E. gracilis* secretes a large number of humic acids

**Fig. 2:**
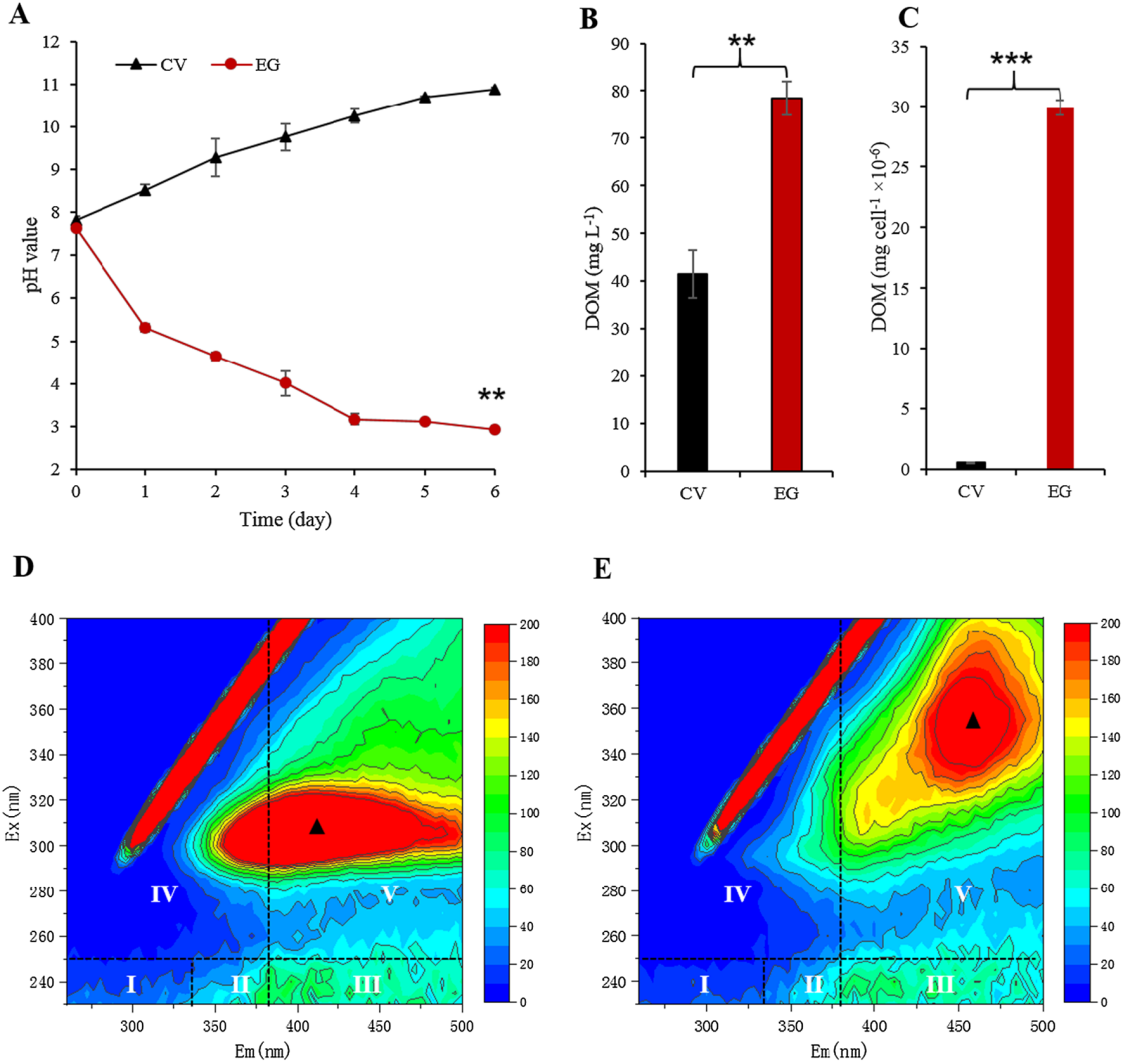
pH and characterization of DOM in the aquatic environment from EG compared to CV. **A** pH value; **B** (quantification by volume) and **C** (quantification by single cells) represent the content of the dissolved organic matter dissolved (DOM) in EG and CV on day 6, respectively; **D**, **E** represent the characterization of DOM via three-dimensional fluorescence excitation-emission matrix (3D-FEEM) spectra (I and II, aromatic proteins, III, fulvic acid-like, IV, soluble microbial by product-like material; V, HA, humic acid) in EG and CV at day 6, respectively; EG represents *E. gracilis*; CV represents *C. vulgaris*; black triangle represents the location of the peak; ** represents *p* < 0.01, *** represents *p* < 0.001; The values represent mean ± SD, where *n* = 3

Generally, microalgae photosynthetic efficiency (e.g., *F*_v_/*F*_m_) is gradually increased, resulting in a large amount of biomass accumulation under photoautotrophic conditions. Most microalgal species can absorb a large amount of CO_2_ dissolved in the aquatic environment, increasing the pH of the aquatic environment [3–5, 29]. This phenomenon was again verified in the CV as the control group in this study, but not in the aquatic environmental pH where EG was cultivated (Fig. 2A). For example, even if the initial pH was set as the same at 7.6, the CV aquatic environmental pH increased from 7.6 to 10.9 on the 6th day of cultivation. In contrast, EG aquatic environmental pH gradually decreased to 2.9. This similar phenomenon of EG has been confirmed by our previous research [9]. This study cultured EG under aseptic and normal photoautotrophic growth conditions (Fig. 1, Additional file 1: Fig. S1). It confirmed that the EG aquatic environmental pH gradually decreased, which was different from the aquatic environmental pH of most microalgae. Therefore, we preliminarily speculated that EG cells were likely to secrete acidic substances that cause the aquatic environmental pH to decrease.

**Fig. 3:**
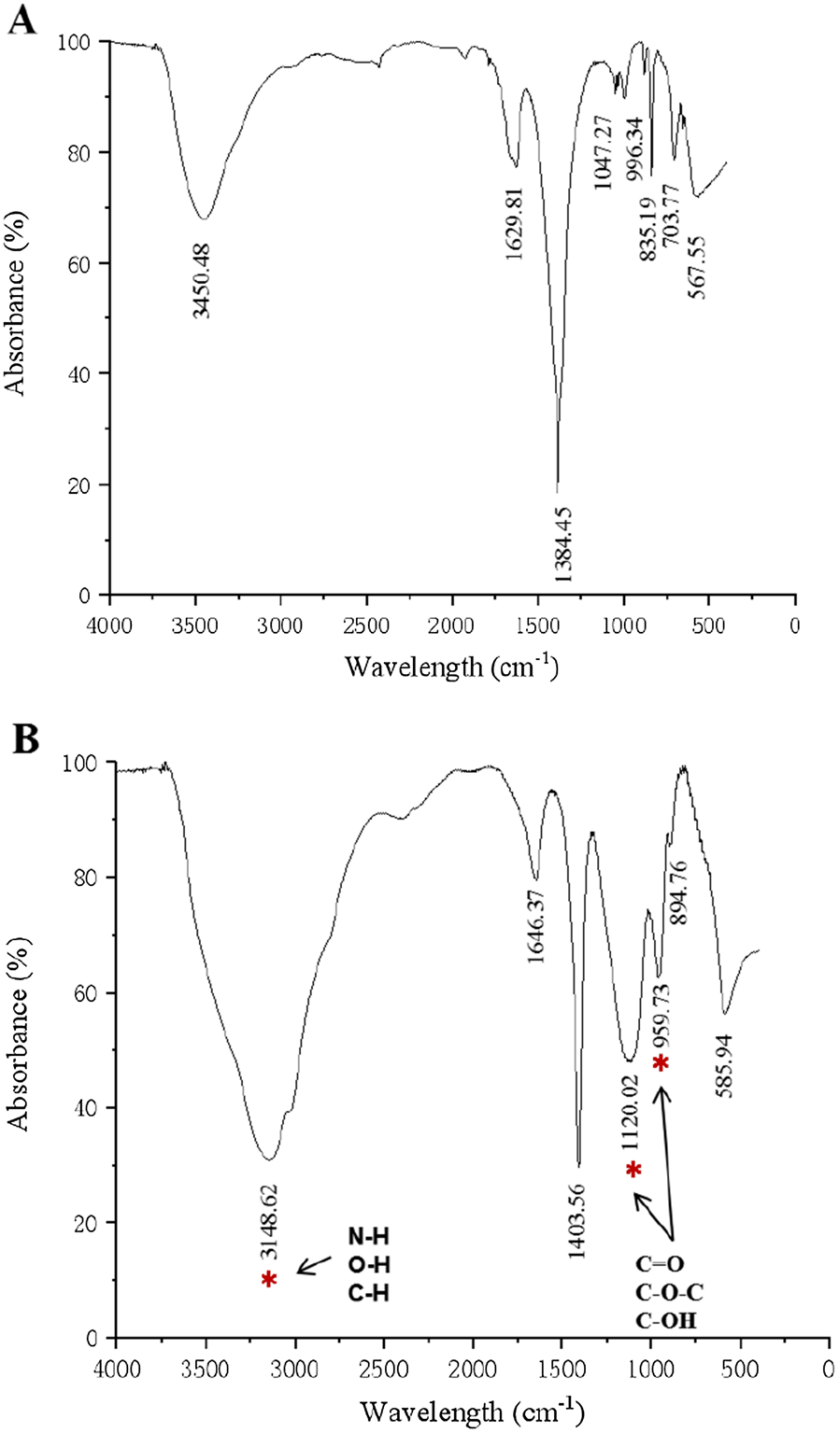
FTIR spectra of the aquatic environment from *E. gracilis* (**B**) compared to *C. vulgaris* (**A**) on day 6

Although EG’s biomass and cell density were lower than CV’s (Fig. 1A, B), the DOM content in the aquatic environment was significantly higher than that of CV. For example, the DOM content of EG (78.5 mg L^−1^) was 1.9-fold that of CV (41.5 mg L^−1^) on day 6 of culture (Fig. 2B, quantification by volume, *p* < 0.01). What is more, the DOM content of EG (29.9 mg cell^−1^ × 10^–6^) was 49.8-fold that of CV (0.6 mg cell^−1^ × 10^–6^) (Fig. 2C, quantification by single cells, *p* < 0.001), indicating that a large amount of DOM was secreted from EG cells. The analysis of 3D-EEM spectroscopy showed that these DOM were mainly composed of humic acid, and EG had a significantly higher fluorescence intensity than that of CV (Fig. 2D, E), indicating that EG secreted a large number of humic acids than that of CV. We used FTIR analysis to reveal their differences to further study the difference in the composition of humic acid secreted by EG and CV. The firm peaks of EG’s aquatic environmental DOM could be detected at wavelengths were 3148.62 (3100–3500 cm^−1^, N–H, O–H, and C–H [18, 21] and 959.73, 1120.02 (900–1200 cm^−1^, C=O, C–O–C, and C–OH [30]) ranges (Fig. 3B) compared to that of CV (Fig. 3A), indicating that these functional groups secreted from EG could readily polymerize to form macromolecular humic acids. These DOM may cause the pH to be reduced in the EG’s aquatic environment. Since EG was a single cell alga without a rigid cell wall like other microalgae, so it may be affected by changes in the external environment and cause the cell to rupture. The substances inside the cell are released into the aquatic environment [9, 22]. However, according to the growth of EG (Fig. 1A–C) and the previous microscopic observations (data not shown), it was unlikely that all metabolites were released into the aquatic environment due to algal cell rupture. Therefore, we need to confirm further which critical metabolites were secreted from the intracellular EG cells.

### pH was reduced by acidic metabolites secretion

**Fig. 4:**
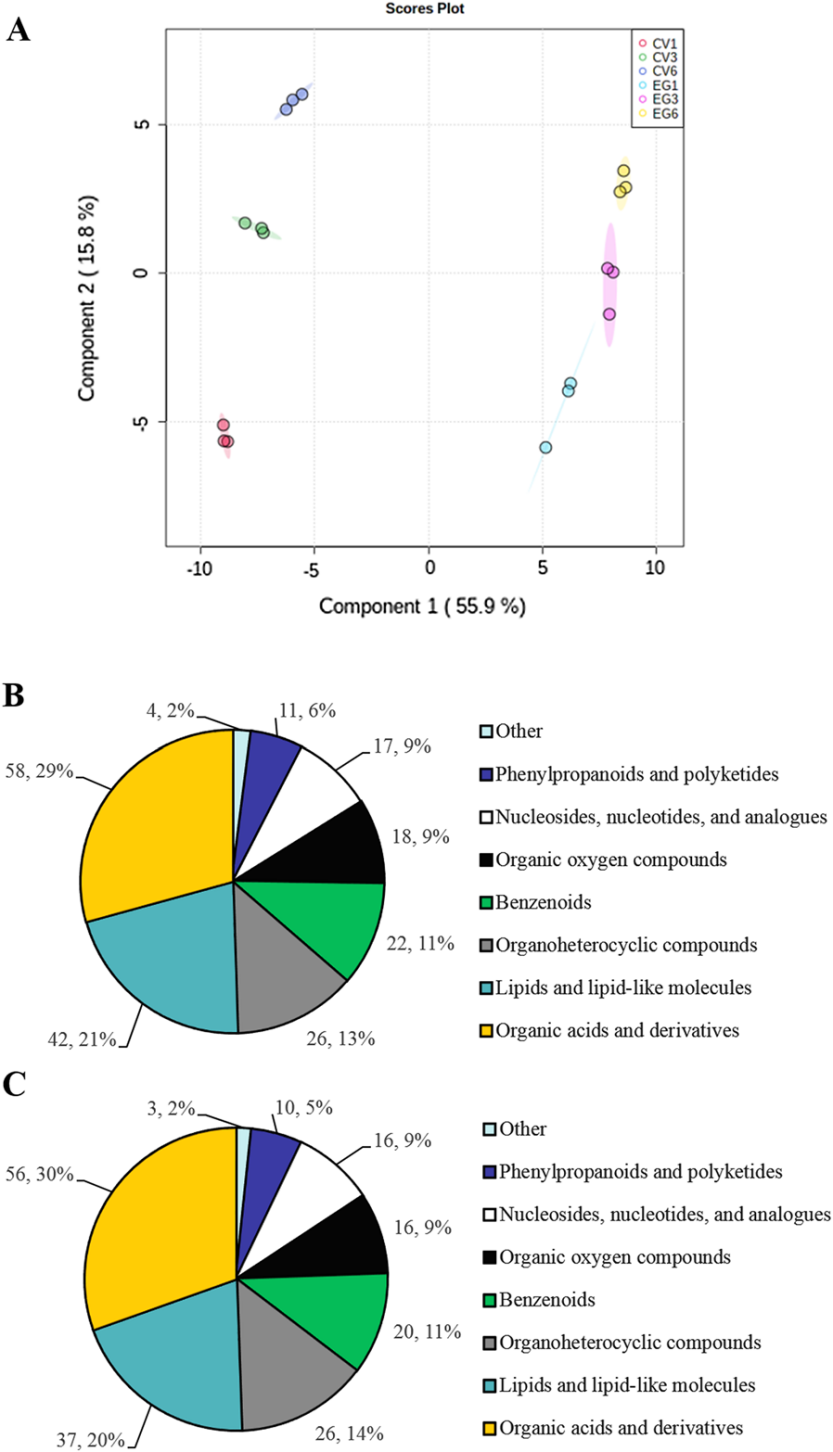
OPLS-DA analysis and composition of DOM in the aquatic environment from EG compared to CV in the negative ion mode*.* **A** The OPLS-DA analysis; **B** the composition of DOM from CV aquatic environment; **C** the composition of DOM from EG aquatic environment; DOM, dissolved organic matter. As the test group, EG1, 3, 6 represents the total metabolite peaks from EG aquatic environment on the 1, 3, and 6 days of cultivation; as the control group, CV1, 3, 6 represents the total metabolite peaks of CV aquatic environment on the 1, 3, and 6 days of cultivation. EG: *E. gracilis*; CV: *C. vulgaris*

To confirm which acidic metabolites were secreted from EG and to analyze how the EG's aquatic environmental pH decreased, the secreted-differential metabolites from EG cells were screened and analyzed via comparative metabolomics methods. 9156 and 10,496 metabolite peaks (Additional file 2: Table S1-NEG-ORG, Table S1-POS-ORG) were detected in negative ion (NEG) and positive ion (POS) modes, respectively. EG and CV could be clearly distinguished under orthogonal-projections-to-latent–structures discriminate (OPLS-DA) analysis (Fig. 4A, Additional file 1: Fig. S2A), indicating that the metabolites were different at the different cultured stages. The metabolites that could be determined in the control group CV in NEG and POS mode were 192 and 239 (Additional file 2: Table S1-NEG-CV, Table S1-POS-CV), and EG were 184 and 294 (Additional file 2: Table S1-NEG-EG, Table S1-POS-EG), respectively. In the NEG mode, these metabolites can be divided into nine categories. Especially, lipids and lipid-like molecules (LLM), organoheterocyclic compounds (OHC), and organic acids and derivatives (OAD) account for the largest proportion of EG and CV metabolites. However, the proportion of EG and CV metabolites was not significantly different (Fig. 4B, C). On the contrary, the proportion of three types of EG metabolites was significantly larger than that of CV in the POS mode (Additional file 1: Fig. S2). It suggested that the three types of metabolites might be secreted from EG cells, causing the aquatic environmental pH to decrease.

**Fig. 5:**
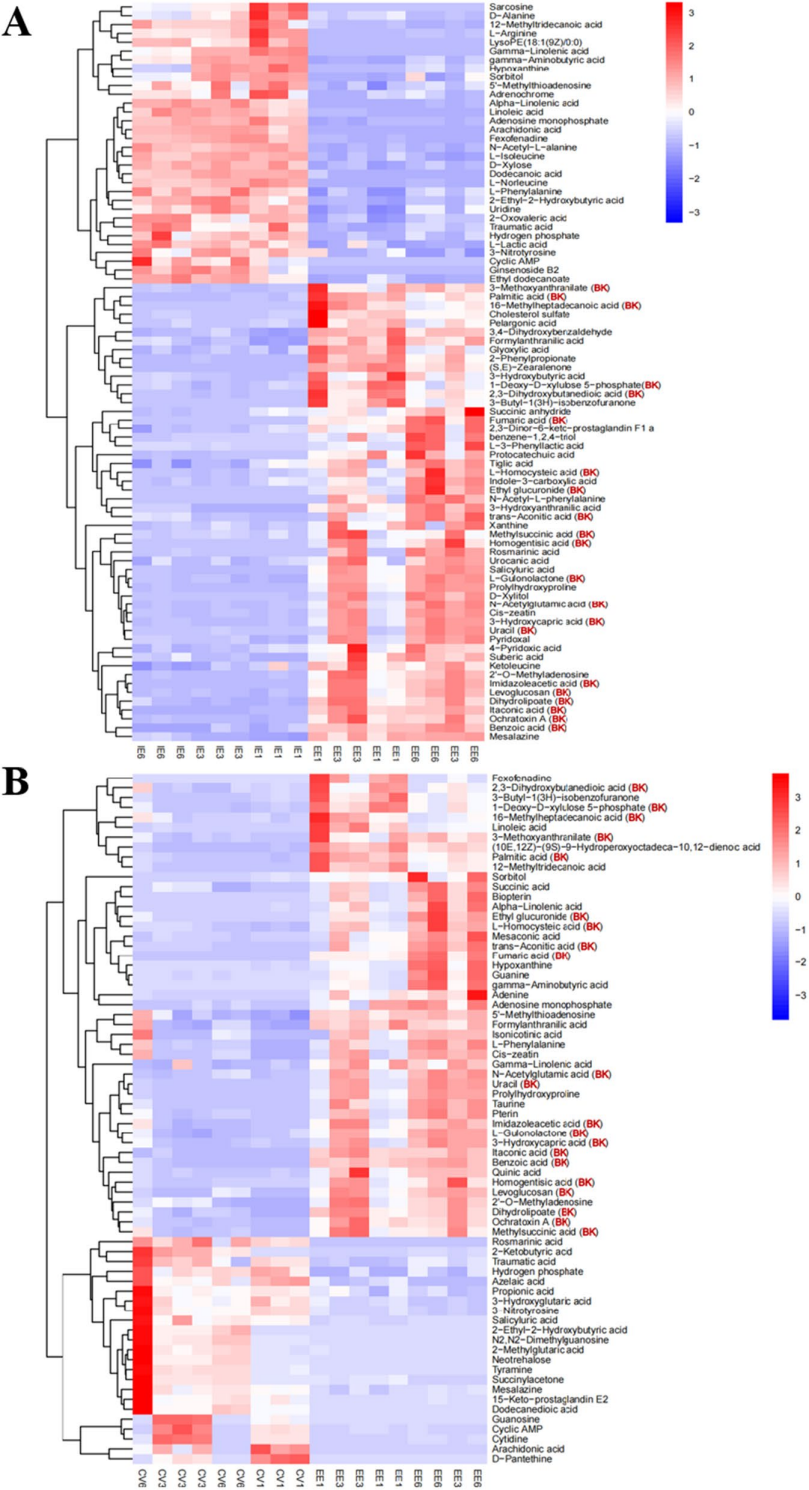
Heat map of differential metabolites. **A** The heat map of *E. gracilis* (EG) differential metabolites between intracellular (IEG) and extracellular (EE); **B** the heat map of differential metabolites from the aquatic environment between *C. vulgaris* (CV) and EG; all metabolites were detected in negative ion mode (NEG mode); BKs, represent candidate biomarkers

To confirm the difference between the critical metabolites secreted from EG cells and the CV in the aquatic environment, we selected the critical metabolites selected by VIP > 1, *P* < 0.05 for cluster analysis. After cluster analysis of the heat map, we found that the relative concentrations of some EG extracellular differential metabolites (EEs) were higher than that of intracellular EG (IEE) (Fig. 5A, Additional file 1: Fig. S3A). Also, the relative concentrations of these EEs were higher than that of extracellular CV (Fig. 5B, Additional file 1: Fig. S3B), indicating that these were candidate biomarker metabolites (BKs), secreted by EG cells.

In addition, it revealed that the distribution of BKs under different pH conditions was different, such on the first day of culture (pH ≈ 5.0), there were 4 metabolites, on the 3rd day (pH ≈ 4.0), there were 12 metabolites, and 4 on the 6th day when the pH is 2.9 (Fig. 5). Similarly, there were similar results in the POS mode (Additional file 1: Fig. S3). It indicated that EG secretes different organic matter under different culture stages and pH conditions. Yoshioka et al. [31] found that when EG was protected from light and under anaerobic conditions, the pH in the medium would affect the secretion of EG metabolites, such as succinic acid, glutamic acid, glutamine, and other substances under acidic conditions (the yield was higher in the pH 3–5). In this study, a similar phenomenon was also found under photoautotrophic conditions. Therefore, it is speculated that EG secretes acidic metabolites, which leads to the pH decrease in the aquatic environment, and negative feedback affects EG secreting metabolites. It is interesting to study the effects of different pH on secreting metabolites of EG under photoautotrophic conditions in the future.

After all metabolites from POS and NEG modes were analyzed, 24 candidate biomarkers (BKs) were confirmed and secreted from EG cells into the aquatic environment (Table 1). Except that the NNP-pKa value of 6-deoxyfagomine was 15.02, slightly higher than that of the control group H_2_O 14. The pKa value of BKs was lower than the acidity value of H_2_O, indicating that the BKs were acidic substances. In addition, BKs were mainly distributed in OAD, LLM, and OHC, which were in line with the results of Fig. 4 mentioned above. In contrast, Zerveas et al. found that the photosynthetic process of microalgae induces pH increase by protons (H^+^) uptake independently in aquatic environment [5], indicating that EG may secreted a lot of H^+^ in the aquatic environment. Finally, it confirmed that the secretion of the acidic metabolites by EG led to the pH decrease in the aquatic environment.

**Table 1:** Summary of 24 candidate biomarkers in the aquatic environment of *E. gracilis*

BK	Super class	XBP-pKa	NNP-pKa	EXP-pKa
1-Deoxy-d-xylulose 5-phosphate	OOC	1.56	1.70	–
2,3-Dihydroxybutanedioic acid	OOC	3.13	2.86	–
Fumaric acid	OAD	3.31	3.09	3.02
*trans*-Aconitic acid	OAD	2.87	3.16	–
Ethyl glucuronide	OOC	3.65	3.45	–
Benzoic acid	BZO	4.15	3.54	4.21
Itaconic acid	LLM	3.48	3.65	3.9
*N*-Acetylglutamic acid	OAD	3.42	3.69	–
Methylsuccinic acid	LLM	3.92	3.86	–
l-Homocysteic acid	OAD	3.58	3.90	–
Homogentisic acid	BZO	4.24	4.17	4.4
3-Hydroxycapric acid	OAD	4.68	4.55	–
Dihydrolipoate	LLM	4.82	4.86	–
3-Methoxyanthranilate	BZO	4.99	4.86	–
Ochratoxin A	PPP	3.54	5.38	–
*cis*-Coutaric acid	OAD	2.48	5.63	–
Palmitic acid	LLM	7.02	5.81	–
16-Methylheptadecanoic acid	LLM	7.08	6.33	–
Imidazoleacetic acid	OHC	5.44	6.56	–
l-Gulonolactone	OHC	7.04	8.42	–
Uracil	OHC	9.40	8.95	9.42
*N*-Methylsalsolinol	OHC	9.92	9.34	–
Levoglucosan	OHC	12.37	10.64	–
6-Deoxyfagomine	OHC	11.63	15.02	–
H_2_O (control)^a^		14.00		

The lower the pKa value, the stronger the acid; XBP- and NNP-pKa represent pKa predicted via XGBoost and Neural Network, respectively; EXP-pKa represents experimental pKa coming from the sub-database of iBonD. For more details, please click http://ibond.nankai.edu.cn. ^a^the pKa value of H_2_O is 14.00 instead of 15.70, according to Silverstein and Heller [46]

*BK* candidate biomarkers, *OOC* organic oxygen compounds, *OAD* organic acids, and derivatives, *LLM* lipids and lipid-like molecules, *OHC* organoheterocyclic compounds, *BZO* benzenoids, *PPP* phenylpropanoids, and polyketides; pKa = − log_10_ Ka, at 25 °C

EG could secrete succinic acid, lactic acid, and amino acids into the aquatic environment [14, 15]. In addition, *E. mutabilis* selectively secreted large amounts of amino acids, polyamine compounds, urea, and some sugars, other organic substances in mine drainages, without fatty acids [13]. However, this study found that EG secreted fewer fatty acids into the aquatic environment under photoautotrophic conditions. In particular, fatty acids account for a relatively large proportion (Fig. 4B, C, Additional file 1: Fig. S2). Therefore, it is speculated that different algae species could selectively secrete metabolites into the aquatic environment under different conditions.

Five hundred million years ago, EG appeared on the Earth. It became highly adaptable in different harsh conditions, such as high UV radiation, heavy metal pollutants, acid mine water, and nutrient deprivation [32, 33]. Halter et al. [13] found that *E. mutabilis* lived in acidic aquatic environments, like acid mine drainages. It could synthesize sufficient oxygen and secrete many metabolites into bacterial communities in the aquatic environment. In addition, Ouyang et al. [34] found that both EG and *Vibrio natriegens* co-cultured in an acidic medium (pH 3.6) promoted the growth and paramylon content of EG. It found that EG secreted a large number of metabolites into the medium. EG provides nutrients for bacteria. In turn, bacteria provide growth-promoting factors for EG. According to these studies, EG may have maintained the genetic characteristics of secreting acidic metabolites to the aquatic environment after long-term evolution, resulting in a decrease in pH, which could selectively inhibit harmful bacteria in an aquatic environment, while acid-resistant, beneficial bacteria may absorb secreted metabolites of EG. Then it provides particular nutrients (such as vitamins B_1_ and B_12_ that could not be synthesized by EG, stably preserved under acidic conditions) to EG, resulting in the balance of the energy and nutrients in the particular aquatic ecosystem.

Heterotrophic yeast acid tolerance mainly relied on combined efforts of Pma1p [35] and V-ATPases [36] to regulate H^+^ permeability. However, the secreted acidic metabolites led to a higher H^+^ concentration in the medium, suggesting that EG lived in an acidic aquatic environment with its cell membranes that were more temporarily impermeable for H^+^. This phenomenon has also been demonstrated in acidophilic algae *Cyanidium caldarium* and *Galdieria sulphuraria* [37, 38]. However, it was still never previously reported whether photosynthetic microalgae, mainly algae EG, had a similar molecular mechanism to regulate intracellular pH, allowing them to live in extreme environment and maintain extreme ecosystems, which deserves future research.

Taken together, this study proposed for the first time that EG could produce organic acids under photoautotrophic conditions verified by comparative metabolomics analysis. Meanwhile, it found that EG can produce acidic substances, of which the BKs were identified to cause the pH decrease in the aquatic environment. At the same time, we have a new understanding of the ecological niche of EG in the aquatic environment.

### Metabolic pathways of critical acidic metabolites

**Fig. 6:**
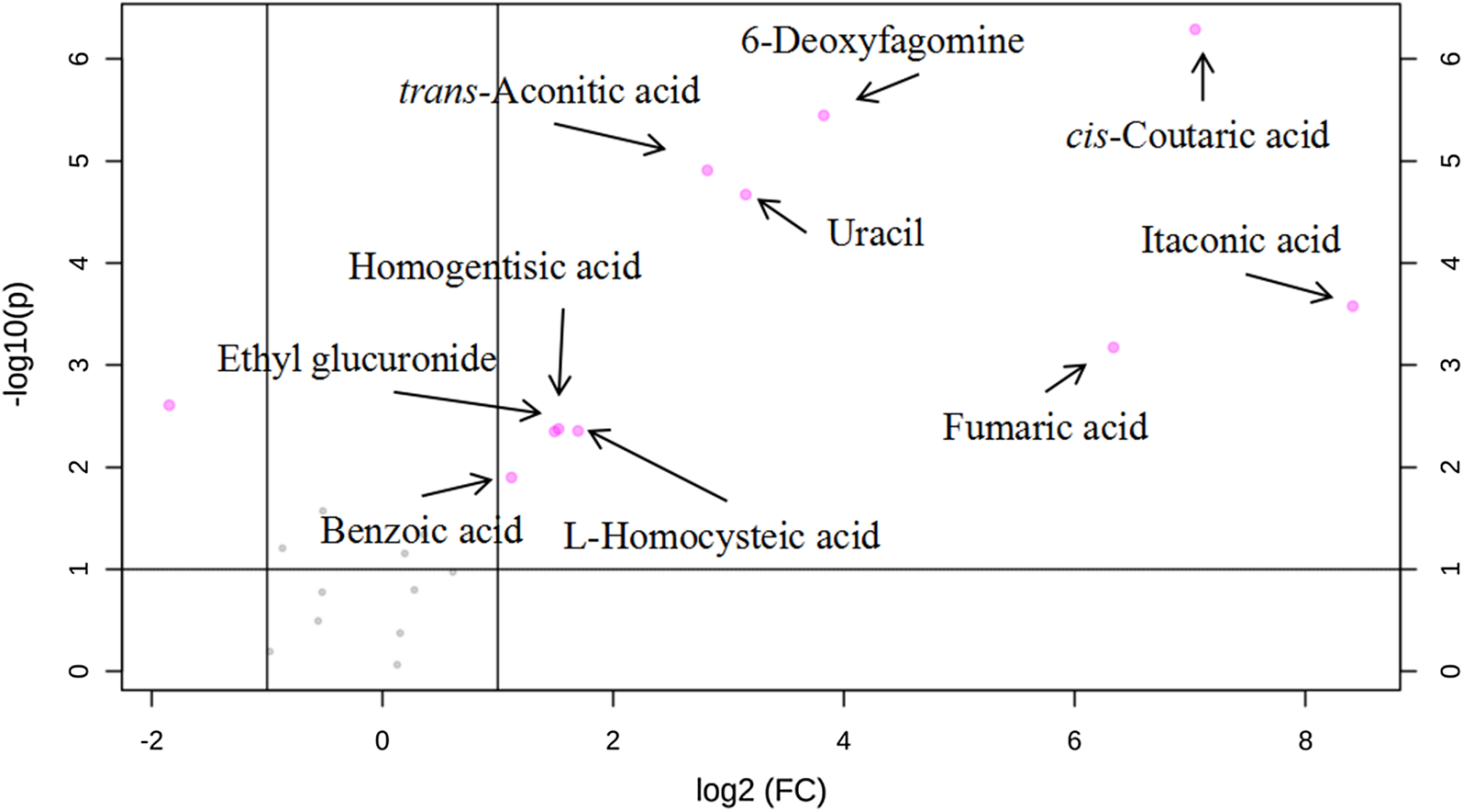
Volcano plot analysis of the BK in *E. gracilis*’ aquatic environment at day 6. BK, candidate biomarkers. The biomarkers were determined via log_2_ (FC) > 1.5, − log_10_ (*p*) > 1

**Fig. 7:**
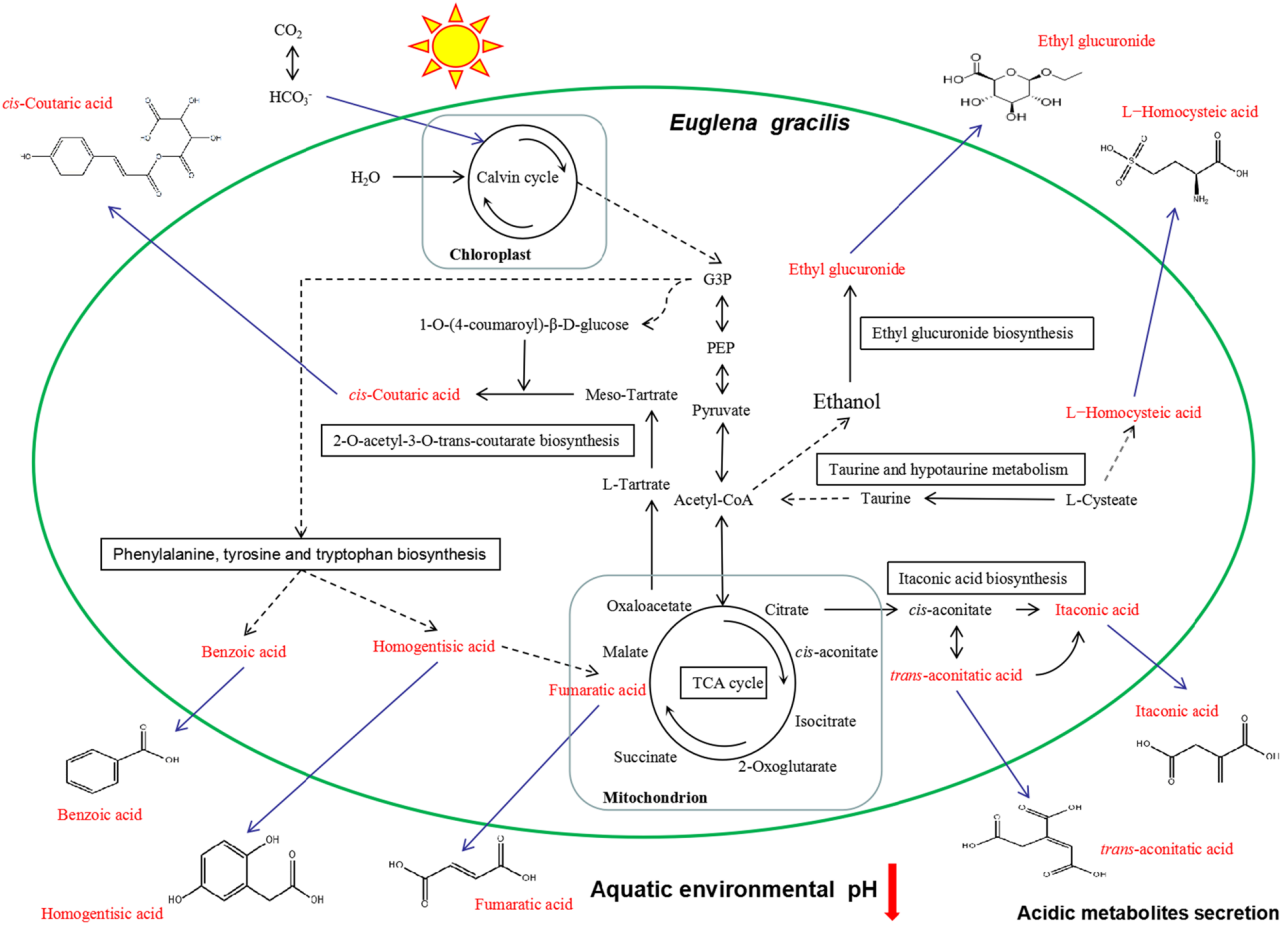
The underlying metabolic pathway of the pH drop in *E. gracilis* aquatic environment. The solid line represents a one-step chemical reaction, and the dotted line represents a multi-step chemical reaction. Red words represent acidic metabolites secretion

As EG was cultivated to the 6th day, the aquatic environmental pH was 2.9, significantly lower than that of CV (pH = 10.9) (Fig. 2A). Therefore, it was meaningful to further study the metabolic pathways of the BKs at this time. In addition to uracil and 6-deoxyfagomine, which have higher pKa values, the relative concentrations of eight critical BKs of EG were significantly different compared to that of CV. Especially, *cis*-coutaric acid, itaconic acid, fumaric acid, and *trans*-aconitic acid (Fig. 6). It indicated that these critical BKs with low pKa values were secreted from EG cells. Those BKs were the most critical factor that caused the aquatic environmental pH to decrease. KEGG analysis showed that the critical BKs were involved in the six critical metabolic pathways, of which itaconic acid biosynthesis was particularly critical (Fig. 7).

Itaconic acid is an unsaturated, dicarboxylic acid with a wide range of applications in the polymer industry and as building blocks for fuels, solvents, and pharmaceuticals [39] and immune activity [40]. Itaconic acid biosynthesis metabolic pathways were only found in fungi [39, 41], marine bivalves [42], and mammalian immune cells [43]. Especially, a large amount of secreted acidic itaconic acid was produced by *Aspergillus terreus* or *Ustilago maydis* with glucose as a carbon source under the heterotrophic fermentation conditions, resulting in a pH decrease [39, 41]. However, this study found for the first time that the photosynthetic autotrophic microalga EG cells using CO_2_ as the carbon source can also secrete relatively high concentrations of itaconic acid into the aquatic environment, that causes the pH to decrease. More interestingly, EG could synthesize organic carbon through photosynthesis and convert it into itaconic acid, reducing growth costs. In the future, we need to elucidate this metabolic pathway, which may provide a new way for environmentally friendly production of itaconic acid. In addition, the biological significance of itaconic acid secretion by EG was also worthy of in-depth study.

This study combined 3D-EEM spectra, FTIR, and metabonomics at different levels to analyze that EG-secreted acidic substances into the aquatic environment under photoautotrophic conditions, resulting in a pH decrease. Consequently, these acidic substances might aggregate together to form humic acid with enormous molecular weight. These findings fill the gaps in scientific research on the secretion of acidic substances from photosynthetically grown EG. Meanwhile, we put forward a new point of view: the increase in the pH of the photosynthetic autotrophic aquatic environment of microalgae was due to not only the high photosynthetic efficiency of microalgae and the absorption of CO_2_ in the aquatic environment but also the microalgae cells not secreting a lot of acidic metabolites. It changed our previous understanding of this discovery. These findings have potential use-value for the control of pH regulation in large-scale microalgae cultivation. For example, EG can be mixed culture with other microalgae to balance the pH of the medium, reduce the cost of artificial pH control, and enhance microalgae biomass. In addition, we have a new understanding of the ecological function of EG in the aquatic environment.

## Conclusion

This study demonstrated that EG caused a decrease in aquatic environmental pH by secreting many acidic metabolites. These metabolites were humic acids composed of N–H, O–H, C–H, C=O, C–O–C, and C–OH. The metabolomic analysis confirmed the critical acidic metabolites secreted by EG and determined its potential metabolic pathways, especially itaconic acid biosynthesis, which was first discovered in microalgae. These studies provide a new understanding of the mechanism of pH changes in the aquatic environment of microalgae culture and the role of microalgae in secreting metabolites to the aquatic environment.

## Materials and methods

### Algal strain and culture conditions

*Euglena gracilis* CCAP 1224/5Z (EG) was purchased from the Culture Collection of Algae and Protozoa (CCAP) and maintained in our laboratory at Shenzhen University. This strain was grown in the photoautotrophic *Euglena* medium (PEM), as described by Wu et al. [9]. In addition, as a control group, *Chlorella vulgaris* FACHB-8 (CV) was obtained from the Freshwater Algae Culture Collection at the Institute of Hydrobiology (FACHB) and grown in BG11 medium [23, 44]. The initial biomass stock (OD_750_ = 0.1) of EG or CV were inoculated to the 800 mL photobioreactors, with 300 mL working solutions for cultivation (Additional file 1: Fig. S1). The initial pH value of the above two culture mediums was adjusted to 7.6 via 3 mol L^−1^ NaOH and 1 mol L^−1^ HCl. The photobioreactors were stirred with 0.2-μm-filtered air (the flow rate was 6 L min^−1^) and illuminated with an LED lamp at a light intensity of 150 μmol photons m^−2^ s^−1^. The temperature of the cultivation remained at 25 °C. The algal biomass, cell density, and aquatic environmental pH were measured every day. *F*_v_/*F*_m_ and metabolomic analysis were performed on days 1, 3, and 6. The characterization of dissolved organic matter (DOM) was analyzed on day 6.

### Algal biomass, cell density, and *F*_v_/*F*_m_

The algal biomass was monitored by measuring the absorbance at 750 nm (OD_750_) and dry weight using a UV–Vis spectrometer (UV2350, UNICO, China) as described [23], respectively. Cell density was monitored using a hemocytometer (Improved Neubauer, USA) every day. The *F*_v_/*F*_m_ ratio was found by dividing the variable fluorescence (Fv) by the maximum fluorescence (Fm), according to the method of Sha et al. [20]. The algae cells were placed in a quartz cube and maintained in the dark for 3 min before measurement of *F*_v_/*F*_m_. The *F*_v_/*F*_m_ ratio for the algae cells was measured at room temperature using a PHYTO-ED fluorimeter (Walz, Effeltrich, Germany).

### Aquatic environmental pH and the characterization of DOM

The aquatic environmental pH value was monitored with a pH meter (pH 30, Clean-Leau Instruments, China). DOM contents were measured using a total organic carbon (TOC) analyzer (Multi N/C 2100, Analytik Jena, Germany). In addition, DOM was quantitated with three-dimensional fluorescence excitation-emission matrix (3D-FEEM) spectrophotometry. Briefly, 3D-FEEM spectra were obtained using a fluorescence spectrophotometer (F-4500, Hitachi, Japan). The excitation (Ex) and emission (Em) slits were set to a bandpass of 5 nm. Ex wavelengths were scanned from 200 to 450 nm, and Em wavelengths were scanned from 220 to 550 nm. All of the 3D-FEEM spectral data were analyzed with Origin Pro 2018 software (www.originlab.com/origin). Fourier transformation infrared spectrometry (FTIR) (Nicolet 6700, Thermo Fisher Scientific, USA) was employed to collect spectra in the range of 400–4000 cm^−1^ at 4 cm^−1^ resolution and 64 scans of the DOM.

### Metabolomics analysis of algae secretion

Intracellular cells and medium of EG and CV were collected, and a metabolomics analysis was performed. The metabolites in the sample were extracted and analyzed according to the method of Wu et al. [23]. The metabolites were detected using ultra-high-performance liquid chromatography coupled with quadrupole time-of-flight mass spectrometry (UHPLC–QTOF-MS). This study detected metabolites after relative standard deviation noise reduction. Next, the missing values were increased by half of the minimum value. An internal standard normalization method was also employed in this data analysis. The final dataset containing the peak number, sample name, and normalized peak area was imported to a SIMCA16.0.2 software package (Sartorius Stedim Data Analytics AB, Umea, Sweden) for multivariate analysis. Data were scaled and logarithmically transformed to minimize the impact of both noise and high variance of the variables. After these transformations, orthogonal-projections-to-latent–structures discriminate analysis (OPLS-DA) was applied. Furthermore, the value of variable importance in the projection (VIP) of the first principal component in OPLS-DA analysis was obtained. It summarizes the contribution of each variable to the model. The metabolites with VIP > 1 and *p* < 0.05 (Student’s t-test) were significantly changed. The pKa values of the candidate biomarkers metabolites (BKs) were analyzed and predicted via the website (pka.luoszgroup.com/prediction) [44, 45]. In addition, the potential metabolic pathway analysis of all BKs was performed via commercial databases, including the KEGG database (www.genome.jp/kegg/), NCBI database (pubchem.ncbi.nlm.nih.gov/compound/), HMDB database (hmdb.ca/), and MetaboAnalyst (www.metaboanalyst.ca/).

### Statistical analysis

Experiments were carried out with biological triplicate cultures. Samples were collected from three microalgal replicates. Data were depicted as mean ± standard deviations (mean ± SD) and statistically analyzed by Student’s *t*-test to investigate differences compared to the control group. A *p*-value less than 0.05 (*p* < 0.05) represents statistically different, while *p* > 0.05 is not statistically significant.

## Supplementary Information

**Additional file 1: Figure S1.** *E. gracilis* and *C. vulgaris* were grown under photoautotrophic and sterile conditions. As the treatment group, EG1, 2, 3 represent three biological replicates of *E. gracilis* (EG); as the control group, CV1, 2, 3 represent three biological replicates of *C. vulgaris* (CV), respectively. The scale bar represents 5 cm. **Figure S2.** OPLS-DA analysis and composition of DOM in the aquatic environment from EG compared to CV in the positive ion mode. A, the OPLS-DA analysis; B, the composition of DOM from *C. vulgaris*’ cultivated media; C, the composition of DOM from *E. gracilis*’ cultivated media; DOM, dissolved organic matter. Composition of DOM in the aquatic environment from *E. gracilis* (EG) compared to *C. vulgaris* (CV) in the positive ion mode. As the test group, EG1, 2, 3 represent three biological replicates of EG; as the control group, CV1, 2, 3 represent three biological replicates of CV, respectively. **Figure S3.** Heat map of differential metabolites. A, the heat map of *E. gracilis* (EG) differential metabolites between intracellular (IEG) and extracellular (EE); B, the heat map of differential metabolites from the aquatic environment between *C. vulgaris* (CV) and EG; All metabolites were detected in positive ion mode (POS mode); BKs, represent candidate biomarkers metabolites.

**Additional file 2.** All metabolites of *E. gracilis* and *C. vulgaris* cells were detected in the NEG and POS modes via comparative metabolomics methods

## Data Availability

All data generated or analyzed in the present study are included in this article and in additional information.

## Abbreviations

EG: *Euglena gracilis*
CV: *Chlorella vulgaris*
DOM: Dissolved organic matter
CCMs: Carbon concentrating mechanisms
RuBisCO: Ribulose-1,5-bisphosphate carboxylase/oxygenase
3D-EEM: Three-dimensional fluorescence excitation-emission matrix
H^+^: Protons
TOC: Total organic carbon
FTIR: Fourier transform infrared
*F*_v_/*F*_m_: Potential maximum photosynthetic capacity
NEG: Negative ion
POS: Positive ion
OPLS-DA: Orthogonal-projections-to-latent-structures discriminate
LLM: Lipids and lipid-like molecules
OHC: Organoheterocyclic compounds
OAD: Organic acids, and derivatives
EEs: Extracellular differential metabolites
IEE: Intracellular EG
BKs: Candidate biomarker metabolites
