# Supplementary material for: Microalgal photoautotrophic growth induces pH decrease in the aquatic environment by acidic metabolites secretion
Source: Biotechnol Biofuels Bioprod. 2022 Oct 26;15:115. doi: 10.1186/s13068-022-02212-z (PMC9608927; doi:10.1186/s13068-022-02212-z)
Supplement: Supplementary file 1 — Additional file 1: Figure S1. E. gracilis and C. vulgaris were grown under photoautotrophic and sterile conditions. As the treatment group, EG1, 2, 3 represent three biological replicates of E. gracilis (EG); as the control group, CV1, 2, 3 represent three biological replicates of C. vulgaris (CV), respectively. The scale bar represents 5 cm. Figure S2. OPLS-DA analysis and composition of DOM in the aquatic environment from EG compared to CV in the positive ion mode. A, the OPLS-DA analysis; B, the composition of DOM from C. vulgaris’ cultivated media; C, the composition of DOM from E. gracilis’ cultivated media; DOM, dissolved organic matter. Composition of DOM in the aquatic environment from E. gracilis (EG) compared to C. vulgaris (CV) in the positive ion mode. As the test group, EG1, 2, 3 represent three biological replicates of EG; as the control group, CV1, 2, 3 represent three biological replicates of CV, respectively. Figure S3. Heat map of differential metabolites. A, the heat map of E. gracilis (EG) differential metabolites between intracellular (IEG) and extracellular (EE); B, the heat map of differential metabolites from the aquatic environment between C. vulgaris (CV) and EG; All metabolites were detected in positive ion mode (POS mode); BKs, represent candidate biomarkers metabolites. [file 13068_2022_2212_MOESM1_ESM.docx]

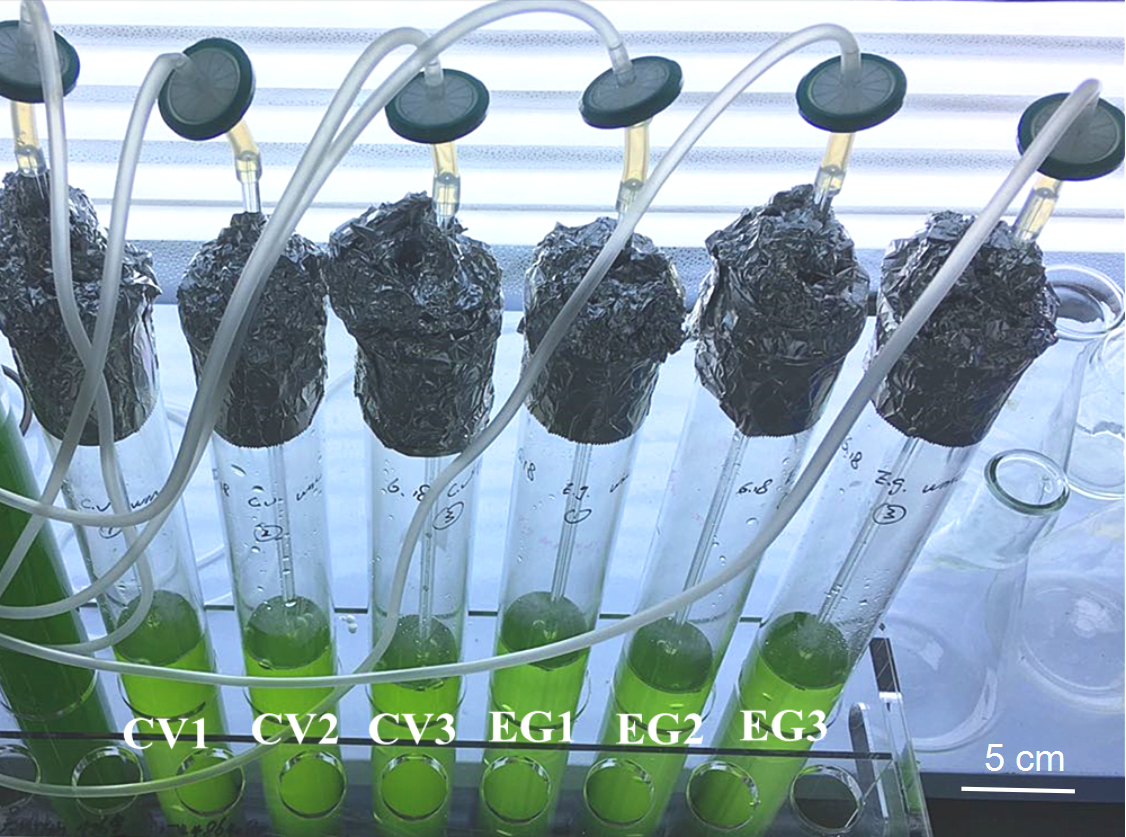


**Figure S1.** *E. gracilis* and *C. vulgaris* were grown under photoautotrophic and sterile conditions. As the treatment group, EG1, 2, 3 represent three biological replicates of *E. gracilis* (EG)*;* as the control group, CV1, 2, 3 represent three biological replicates of *C. vulgaris* (CV), respectively. The scale bar represents 5 cm.


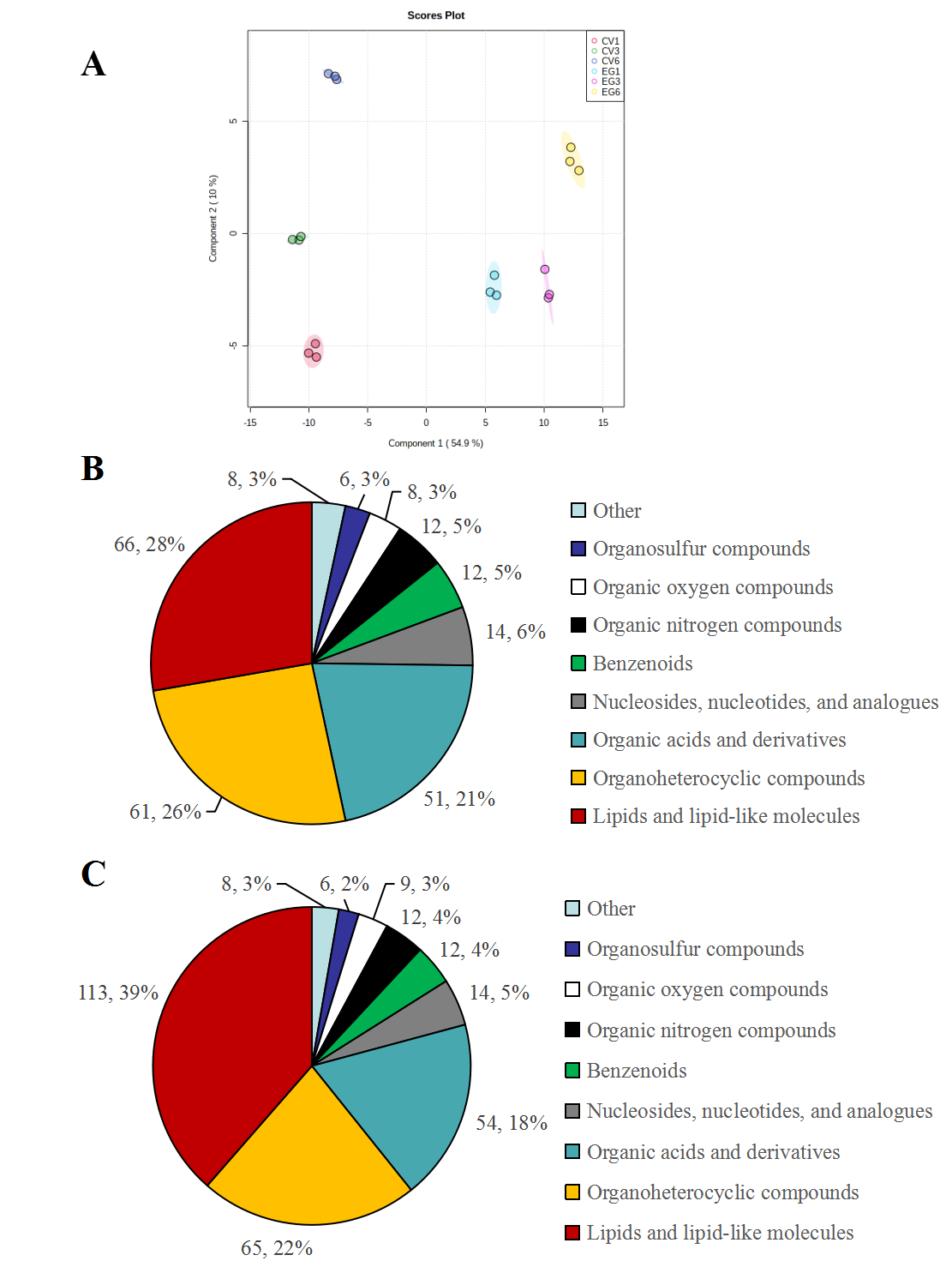


**Figure S2.** A, the OPLS-DA analysis; B, the composition of DOM from *C. vulgaris*’ cultivated media; C, the composition of DOM from *E. gracilis*’ cultivated media; DOM, dissolved organic matter. Composition of DOM in the aquatic environment from *E. gracilis* (EG) compared to *C. vulgaris* (CV) in the positive ion mode. As the test group, EG1, 2, 3 represent three biological replicates of EG*;* as the control group, CV1, 2, 3 represent three biological replicates of CV, respectively.


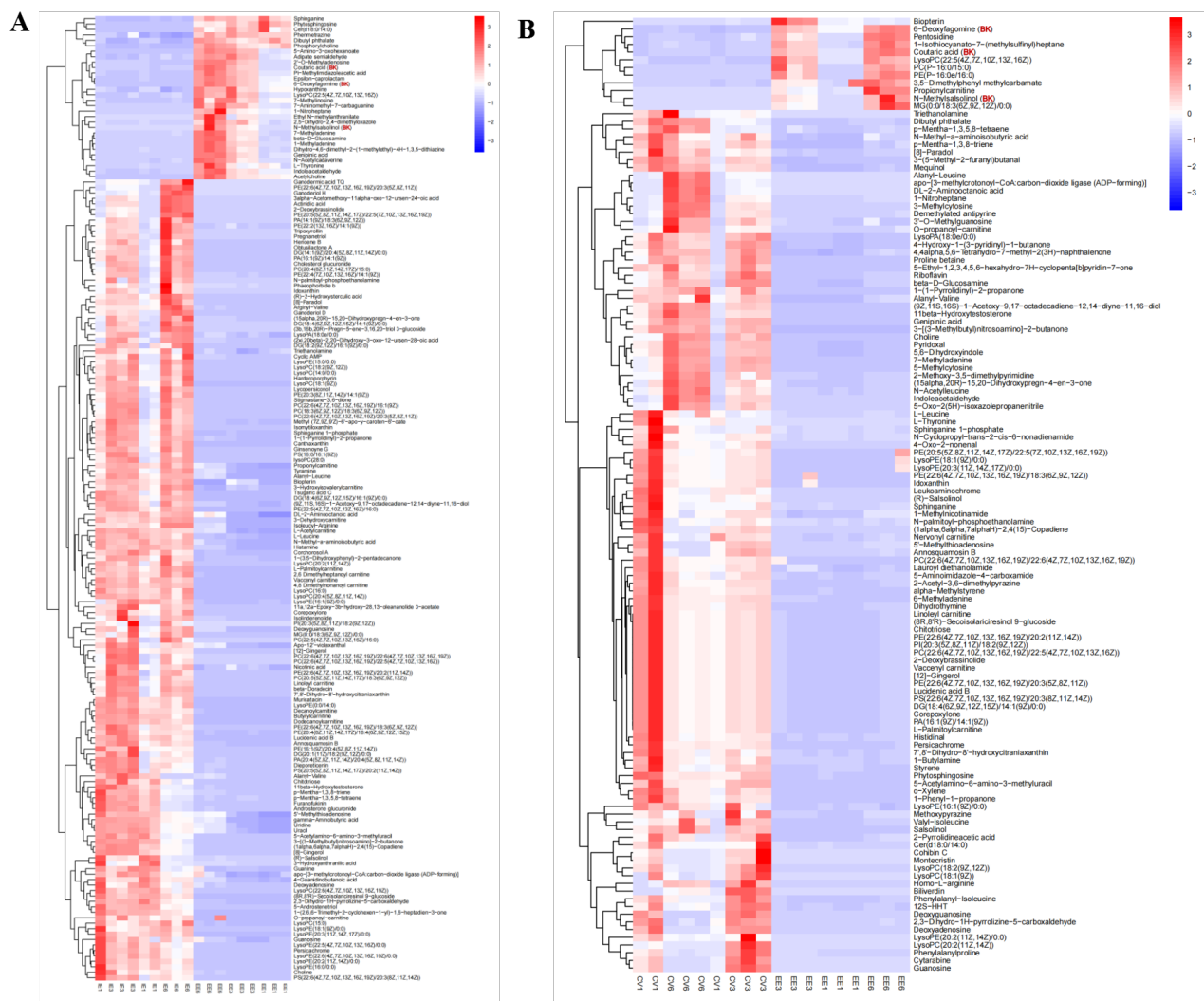


**Figure S3.** Heat map of differential metabolites. A, the heat map of *E. gracilis* (EG) differential metabolites between intracellular (IEG) and extracellular (EE); B, the heat map of differential metabolites from the aquatic environment between *C. vulgaris* (CV) and EG; All metabolites were detected in positive ion mode (POS mode); BKs, represent candidate biomarkers metabolites.
